# Managing Aggressive Behaviour in Healthcare: Balancing of Patients and Staff Interests

**DOI:** 10.21315/mjms2024.31.3.20

**Published:** 2024-06-27

**Authors:** Yusrita Zolkefli

**Affiliations:** 1 PAPRSB Institute of Health Sciences, Universiti Brunei Darussalam, Brunei Darussalam; 2 School of Health in Social Science, The University of Edinburgh, Edinburgh, United Kingdom

**Keywords:** restraint, ethics, aggression, safety, violence, delivery of health care

## Abstract

The incidence of aggression within healthcare environments has exhibited a discernible rise. As a response, restrictive measures, including restraints, are enforced. Due to the safety and ethical concerns associated with using restraint, de-escalation measures are regarded as the most efficient course of action. The paper posits that it is critical to identify the causes of aggression before employing restraint through a multidisciplinary risk assessment. In addition, the reasonableness and proportionality of administering restraint must be carefully measured. The significance of cultivating a therapeutic and compassionate environment is emphasised. The paper will exclusively examine physical restraint as a form of restriction intervention.

## Introduction

Aggressive behaviours continue to be an ongoing threat within healthcare settings, eliciting concerns regarding the safety and well-being of both patients and the healthcare professionals administering the care (1). Aggression consists of several aspects. Verbal aggression includes threats, profanity, loud noises, shouting, insults, foul language and swearing, whereas physical aggression consists of striking, grasping, punching and swinging. Aggression often encompasses the deliberate intention of causing harm to the target, while violence is an extreme form of aggression. The causes of aggressive behaviour are associated with patient, staff and environmental variables. For example, inexperienced staff and interactions between patients and staff may provoke and intensify aggressive behaviour in patients (2). Patient-related and environmental factors, such as age, gender and clinical diagnoses, as well as inconsistent policies and guidelines, may contribute to aggression. Such a challenging atmosphere, therefore, complicates the fundamental goals of healthcare, which are the recovery and safety of patients. In response, restrictive interventions are frequently implemented by healthcare professionals. Physical restraint, mechanical constraint, seclusion and forced medication are examples of these interventions. Such an intervention has inevitably led to an ethical dilemma, psychological distress experienced by staff and patients, and the possibility of harm being done to both sides. Some patients, for example, have reported suffering negative emotional responses after being physically restrained for an extended period (3). Because of this, there has been a concerted effort on a global scale to eliminate or significantly reduce the interventions, including physical restraint. (4). Yet, the interventions continue to be widely used to manage aggressive conduct despite the evidence that it reportedly has no therapeutic benefit. This appeal has resulted in ongoing debates.

### An Ethical View of the Restraint

Restrictions are often put in place to protect the safety of staff, patients and other individuals (5). This aligns with the ethical responsibility of maintaining a safe environment in healthcare settings. Moreover, the right of healthcare professionals to carry out their duties in a safe setting is recognised as an important priority. While the need for restrictive interventions to safeguard the well-being of both patients and staff has been a subject of debate, it is generally acknowledged that they often result in undesirable outcomes (6). Additionally, there have been reports from staff members regarding diminished therapeutic interactions with patients, anxiety and job dissatisfaction (7). Added to that, these procedures have been reported to be dehumanising, upsetting, puzzling and agonising by individuals undergoing the interventions (8). Furthermore, fatalities may sometimes occur, which is even more alarming (9).

On the other hand, more patients are concerned about the responses taken by healthcare professionals when the purpose of using restraint measures is nebulous. This is especially true when there is a lack of staff, when staff convenience is prioritised over patient dignity and when the interventions are misused. In addition, when staff overly resort to restraint, ethical questions are raised regarding the appropriateness of such measures. This kind of restraint goes against the principles of doing good (beneficence) and avoiding harm (non-maleficence). The tension that exists between respecting the patient’s autonomy and safeguarding their safety is brought to light by this observation (10).

Along with this, patient care may be compromised when staff label certain patients as ‘difficult’ (11), leading to their isolation, especially during times when they are most vulnerable (12). Interventions in certain situations can also potentially worsen the incidents, causing increased violence (13). In addition, it is essential to prioritise the preservation of their dignity rather than focusing disproportionately on the patient’s clinical diagnosis, particularly in cases when the patient has a mental diagnosis (14). Hence, the notion of restraint demands an appropriate compromise between preserving the patient’s autonomy and ensuring their safety (15). Yet, despite the potential negative consequences, physical restraint has been deemed ethically acceptable as long as it remains focused on protecting patient safety and reestablishing the patient’s autonomy. Utilitarianism, for instance, places key importance on the welfare of all individuals to promote overall well-being. Consequently, it is thought that exercising control under extraordinary circumstances was essential to protect patients and others from the harmful consequences of aggressive behaviour (16).

### Navigating the Aggressive Behaviour

Healthcare professionals encounter uncertainty when faced with aggressive behaviour. Hence, it is essential to examine three pragmatic approaches when deciding the best course of action for managing the incident. Firstly, it is essential to determine the underlying factors contributing to the aggressive behaviour exhibited by the patient. Situations may usually be resolved by identifying the root causes, recognising the needs of patients and promptly meeting those needs. Healthcare professionals are required to have the ability to distinguish between aggression that originates from medical conditions, such as severe hypoglycemia leading to mental confusion or hallucinations, and psychiatric disorders, such as psychotic illnesses resulting in agitation or abusive behaviour that arise from factors unrelated to mental health. Erroneous clinical assessments or misinterpretation of the underlying factors contributing to the aggression can result in premature decisions or inadequate interventions. Prejudiced attitudes may arise while addressing such behaviour, especially among those with a background of psychiatric conditions (17).

Meanwhile, it is important for professionals to recognise that their clinical assessments can be influenced by factors such as patient gender and ethnic background (18). Instead, healthcare professionals are advised to prioritise the development of relationships, engage in negotiation and practise effective communication to handle the situations (1). Staff members are strongly advised to maintain emotional composure during and after the aggressive incident (19). The intervention or response must be properly focused to address the patient’s particular needs, regardless of the patient’s actions or the resulting tension.

Secondly, it is crucial for healthcare professionals to conduct an impartial assessment of potential risks to prevent any form of aggressive or violent behaviour and establish a safe treatment environment promptly and without using force (20). The primary goals of risk assessment are to reduce the need for restrictive intervention, determine the appropriateness of restraints, and facilitate anticipation of impending aggression and perceived threats. When evaluating threats in high-risk settings, healthcare professionals conscientiously apply the principles of necessity. In doing so, they must carefully assess the reasonableness and proportionality of the restraint. Reasonability dictates when the degree of aggression threat exceeds what is considered acceptable and when restraint is absolutely necessary (21). At the same time, reasonable approaches strive to refer and seek appropriate assistance from various teams of specialists so that any potential use of restraints is effective and proportionate with the perceived risks. As an example, when psychiatric illness patients manifest aggressive behaviour in medical environments, a risk assessment may also involve the participation of a multidisciplinary liaison team comprised of psychiatric nurses and psychiatrists. The assessment is crucial to guarantee that the restricted intervention is customised to meet the patient’s specific needs and complies with the institution’s guidelines.

Following this, the most effective approach to managing aggression can be instigated. To manage aggressive conduct effectively, it is recommended that non-coercive measures be used as an initial approach (22). These approaches include measures for de-escalation, time-outs, increased supervision and medication administered in collaboration with the patient. However, other proactive measures may be considered if de-escalation is unsuccessful in preventing aggression. To put it another way, as the amount of intervention restriction goes from minimal to maximal, it may become justifiable to use a temporary physical restraint that safeguards patients and restores their autonomy (23). Protocols and guidelines mandate the use of restraint in exceptional circumstances, and its appropriate application is decided by a multidisciplinary team with professional expertise in therapeutic restraint (24).

## Conclusion

Continued efforts to protect hospital staff and patients from potential risks caused by aggressive behaviour are of the highest priority. Given the potential for harm to patients and staff, it is crucial to acknowledge that aggressive behaviour may serve as a sign of unaddressed needs before anything else. To address any ethical tension associated with it, it is necessary to establish a care plan that integrates clear management protocols with a comprehensive risk assessment from a multidisciplinary team to strike a balance between the interests of the staff and the patients. This will enable staff to respond fairly and impartially to aggressive behaviour.
